# Impact of adipocytes on ultrasound evaluation of parathyroid adenomas

**DOI:** 10.1007/s10396-024-01511-2

**Published:** 2024-12-28

**Authors:** Tomoko Fujimoto, Mitsuyoshi Hirokawa, Ayana Suzuki, Maki Oshita, Hiroyuki Yamaoka, Makoto Fujishima, Naoyoshi Onoda, Akira Miyauchi, Takashi Akamizu

**Affiliations:** 1 Department of Clinical Laboratory, Kuma Hospital, 8-2-35 Shimoyamate-Dori, Chuo-Ku, Kobe, Hyogo 650-0011 Japan; 2 Department of Diagnostic Pathology and Cytology, Kuma Hospital, Kobe, Hyogo 650-0011 Japan; 3 Department of Internal Medicine, Kuma Hospital, Kobe, Hyogo 650-0011 Japan; 4 Department of Surgery, Kuma Hospital, Kobe, Hyogo 650-0011 Japan

**Keywords:** Ultrasound, Parathyroid adenoma, Lipoadenoma, Two-tone pattern

## Abstract

**Purpose:**

Parathyroid lipoadenomas are difficult to recognize preoperatively; hence, they may remain undetected. Difficulty in recognition is thought to be due to the adipocytes present in the tumor. This study aimed to clarify the impact of adipocytes as a component of parathyroid adenomas on ultrasound evaluation.

**Methods:**

Eighteen parathyroid adenoma cases, in which the adipose tissue accounted for more than 10% of the tumors, were included in this study. Of these, five were consistent with lipoadenomas. Twenty-five consecutive patients with parathyroid adenoma without adipocytes were used as controls.

**Results:**

Ultrasonography revealed a lipoadenoma detection rate of 20.0%. This increased to 80.0% at re-examinations performed after obtaining information from other imaging modalities. Compared with parathyroid adenoma cases with no adipocytes or few adipocytes, the frequencies of ill-defined margins, iso- and/or hyperechogenicity, heterogeneous consistency with a two-tone pattern, poor vascular flow, no polar artery, and no hyperechoic line were significantly higher in parathyroid lipoadenoma cases. The hyperechoic and isoechoic areas in tumors with a two-tone pattern correspond to adipocyte- and parathyroid cell-rich areas, respectively. The lipoadenoma tumor sizes measured using ultrasound tended to be smaller than the actual sizes.

**Conclusions:**

The characteristic ultrasound findings of lipoadenomas were clearly different from those of parathyroid adenomas with or without adipocytes. We believe that our findings may contribute to an increased detection rate of lipoadenomas and allow us to consider them in the differential diagnosis.

## Introduction

Parathyroid adenomas are benign neoplasms derived from parathyroid parenchymal cells and are mostly associated with hyperparathyroidism. Histologically, the tumors are composed of chief, transitional, oncocytic, and water-clear cells. Stromal adipocytes are either absent or scattered. Parathyroid lipoadenoma, containing considerable amounts of adipocytes, is a rare type of parathyroid adenoma with a prevalence of 0.2% in primary hyperparathyroidism [1]. To date, fewer than 100 cases of parathyroid lipoadenoma have been reported [1, 2]. This tumor is characterized by an increase in both parathyroid parenchymal cells and adipocytes. The proportion of adipocytes in parathyroid lipoadenomas varies between studies, ranging from 20 to 50% [3–5]. The latest World Health Organization (WHO) classification defines parathyroid lipoadenomas as those with more than 50% of the volume comprising adipocytes [1, 5]. The tumor is distinguished from normal parathyroid glands by increased volume, the presence of hyperparathyroidism, a significant drop in intraoperative parathyroid hormone (PTH) levels, and evidence of postoperative biochemical cure [5].

Among the preoperative imaging modalities for parathyroid adenomas, ultrasonography (US) is a quick, well-tolerated, and cost-effective first-line imaging technique. The sensitivity and specificity of US for parathyroid adenomas is 76–79% and 96%, respectively [6]. Parathyroid adenomas are commonly hypoechoic [7–9]. However, lipoadenomas frequently present as hyperechoic nodules and are difficult to recognize preoperatively; therefore, they may go undetected [2–4, 7, 8, 10–17]. The difficulty in recognition is thought to be due to the fat cells present within the tumor, but there have been no detailed reports on their impact on ultrasound images. Hence, this study aimed to shed light on the impact of adipocytes as a component of parathyroid adenomas on US evaluation.

## Materials and methods

### Study population

The study protocol was reviewed and approved by the Institutional Review Board of Kuma Hospital (approval number: 20231012-4), and the study was conducted in accordance with the 1964 Declaration of Helsinki and its amendments or comparable ethical standards. All the study participants provided informed consent. In this retrospective study, we reviewed the surgical pathology data of 1,812 patients who underwent parathyroidectomy at Kuma Hospital between January 2010 and December 2022, and we selected 1,703 cases with a histological diagnosis of parathyroid adenoma. Among them, 18 patients (1.08%) with more than 10% adipocytes in tumor volume on microscopic preparations were included in this study. Nine of these were previously reported by our coauthors in a separate study [3]. Twenty-five consecutive patients with a histological diagnosis of parathyroid adenoma without adipocytes, resected between October 2022 and December 2022, were also examined as controls. No participants received calcium-sensing receptor (CaSR) agonists preoperatively.

### US and data collection

US was performed using the APLIO 80 SSA-770A (Canon Medical Systems Co., Ltd., Otawara, Japan) and APLIO 500 TUS-A500 (Canon Medical Systems) system with a PLT-805AT probe (5 and 12 MHz, Canon Medical Systems), PLT-1005BT probe (5 and 14 MHz, Canon Medical Systems), or PVT-712BT probe (4–11 MHz, Canon Medical Systems). Clinical data were obtained from patients’ medical records at Kuma Hospital. Serum intact PTH and calcium levels were taken from measurements obtained immediately before surgery. We retrospectively examined the US reports and photographs saved in medical records. Nineteen clinical technologists who specialized in thyroid ultrasonography performed the examinations. If the findings were debatable, the clinical technologists reported the results after consulting a senior technologist. In four cases, photographs were not available. The US findings examined included tumor size, shape, margin, composition, echogenicity, calcification, blood flow signal, and the hyperechoic line between the thyroid and the tumor. Blood flow signals were evaluated using power Doppler and/or advanced dynamic flow imaging. Tumor size was measured in three directions, and the largest diameter was used as the tumor size. Tumor compositions were classified as solid, predominantly solid, predominantly cystic, and cystic [18]. Internal echogenicity was evaluated based on a normal thyroid gland. Histological examination was performed using representative hematoxylin and eosin-stained preparations. Tumor size was also measured on microscopic preparations, and the results were identified as the accurate size of the tumor. Parathyroid adenomas and lipoadenomas were classified into four categories according to the proportion of adipose tissue contained in the tumor: group 1, ≤ 10%; group 2, from 11% to 30%; group 3, from 31 to 50%; and group 4, ≥ 51%. Of the 18 cases associated with adipocytes, six, seven, and five were classified into groups 2, 3, and 4, respectively. Group 1 was considered a typical parathyroid adenoma, while group 4 met the diagnostic criteria for a lipoadenoma.

### Statistical analysis

Data are presented as medians with interquartile ranges or numbers (percentages). The Kruskal–Wallis and Pearson’s χ2 tests were used for statistical analyses, and a *p*-value of less than 0.05 was considered a statistically significant difference.

## Results

### Clinical findings

Table 1 shows the clinical findings of the parathyroid adenoma and lipoadenoma cases. Patients with parathyroid lipoadenomas (group 4) had a median age of 66.0 years (range: 63.0–84.0 years), and included two men and three women. The other groups (groups 1, 2, and 3) also predominantly consisted of women. No significant differences in age or sex were observed between the groups. The intact PTH level increased preoperatively, and postoperative biochemical cure was demonstrated in all cases. Medians of intact PTH and serum calcium levels among patients in group 4 were 137.0 pg/mL and 10.8 mg/dL, respectively. However, they did not differ significantly from those of the other groups. There were no predictive sites in any group regarding the location of the lesions. Lipoadenoma detection rates at the first visit were significantly lower in groups 3 (14.3%) and 4 (20.0%) than those in groups 1 (88.0%) and 2 (33.3%) (p < 0.0005). In repeated preoperative examinations performed after obtaining information from other imaging modalities, the detection rate in group 4 increased to 80.0%; however, this was lower than that in group 1 with 100%. None of the patients were suspected to be lipoadenoma, lipoma, or lymph node. A lesion in one case in group 4 was interpreted as an ectopic thymus.

**Table 1:** Clinical findings in parathyroid adenoma and lipoadenoma cases

	Adenoma			Lipoadenoma	*p-*value
	Group 1 (n = 25)	Group 2 (n = 6)	Group 3 (n = 7)	Group 4 (n = 5)	
Age; years (median)	26–89 (57.0)	55–84 (71.5)	42–80 (56.0)	63 – 84 (66.0)	NS
Men: Women	4:21	0:6	2:5	2:3	NS
Intact PTH pg/mL (median)	53–527 (111.0)	93–171 (135.5)	10–224 (136.0)	129–186 (137.0)	NS
Calcium mg/dL (median)	10.1–13.0 (11.0)	10.2–11.4 (10.9)	8.6–12.2 (11.0)	9.7–12.1 (10.8)	NS
Location					
Upper right	6 (24.0%)	0 (0%)	1 (14.3%)	1 (20.0%)	NS
Upper left	7 (28.0%)	2 (33.3%)	4 (57.1%)	0 (0%)	
Lower right	4 (16.0%)	2 (33.3%)	1 (14.3%)	1 (20.0%)	
Lower left	8 (32.0%)	1 (16.7%)	1 (14.3%)	3 (60.0%)	
Intra-thyroid	0 (0%)	1 (16.7%)	0 (0%)	0 (0%)	
Detection rate					
First visit	88.0%	33.3%	14.3%	20.0%	< 0.0005
Preoperative	100%	50.0%	71.4%	80.0%	NS

PTH, parathyroid hormone; NS, not significant

### US findings

**Fig. 1:**
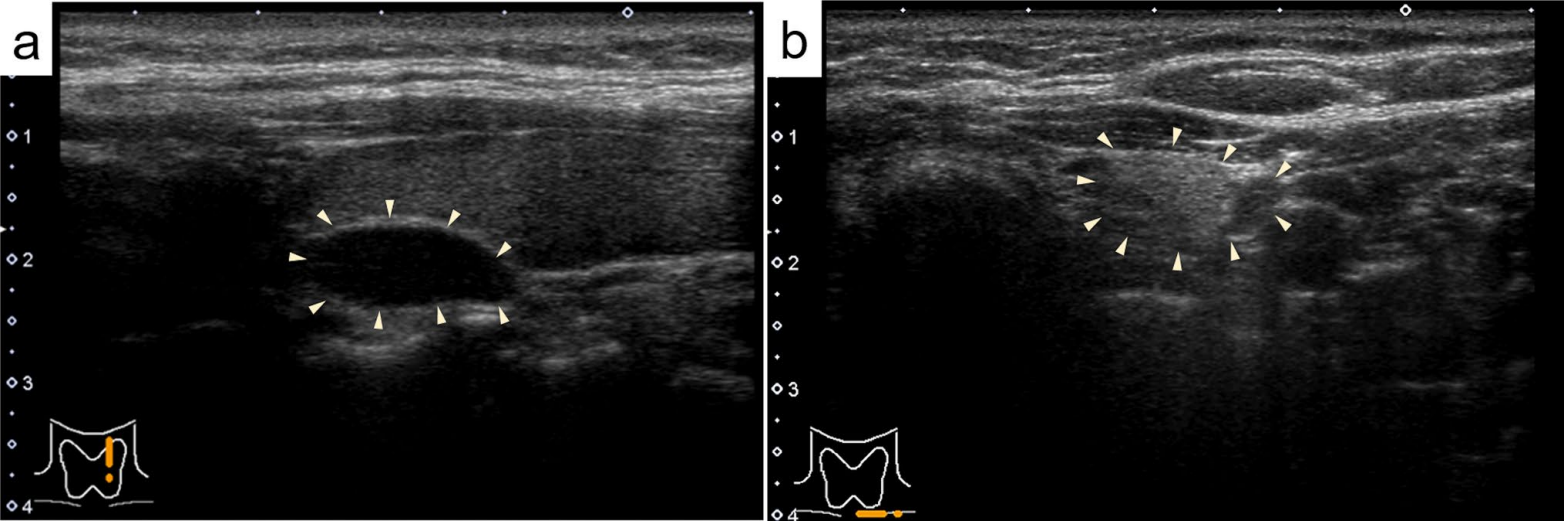
**a** (Group 1): The tumor is well-defined, hypoechoic, and shows a hyperechoic line at its border with the thyroid. **b** (Group 4): The tumor is ill-defined, heterogeneous, and hyperechoic. The triangular arrows indicate the margins of the tumor (B-mode)

**Fig. 2:**
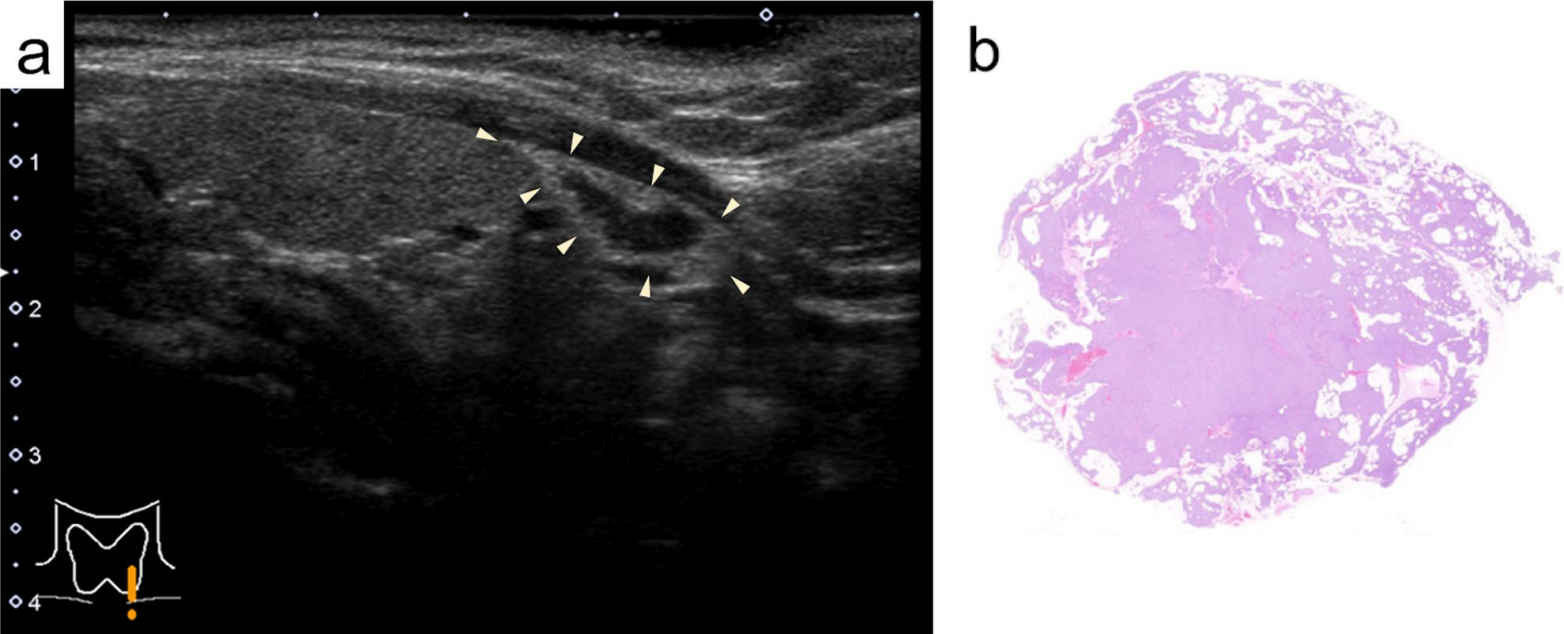
**a**: A parathyroid adenoma showing a two-tone pattern (Group 2) (B-mode). **b**: The hypoechoic and isoechoic areas correspond to adipocyte- and parathyroid cell-rich areas, respectively (hematoxylin and eosin-stained preparation)

**Fig. 3:**
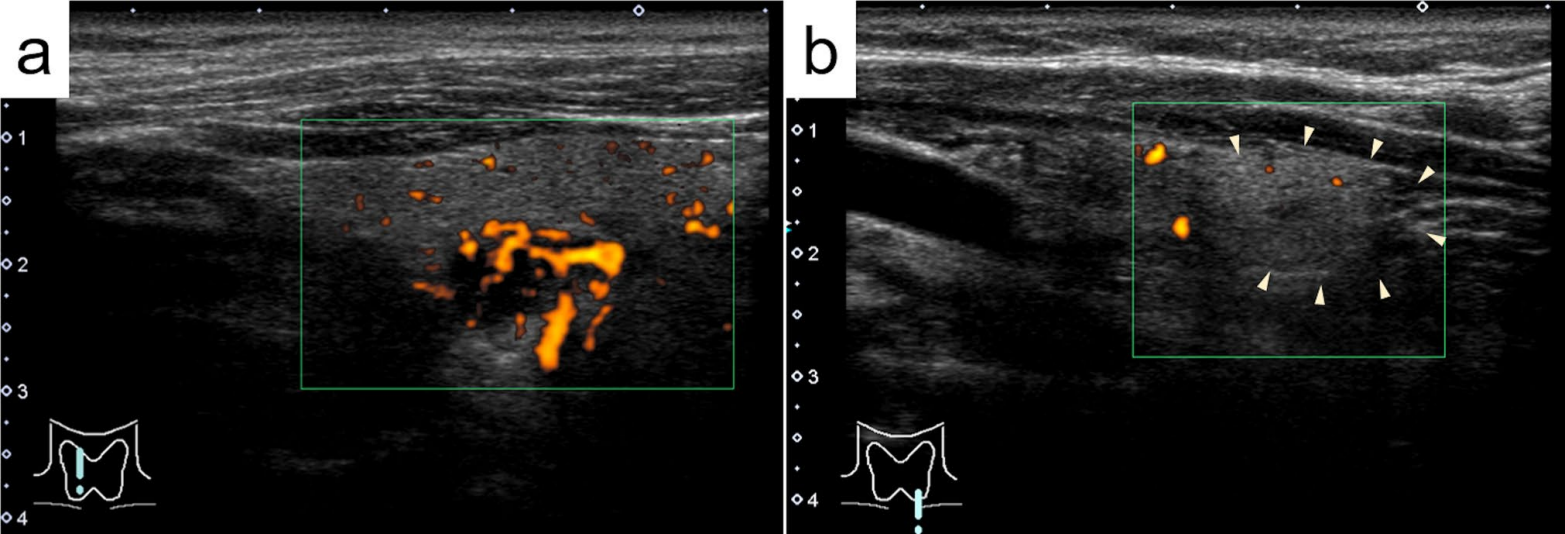
**a** (Group 1): A blood flow signal is present within the nodule. Note the polar artery. **b** (Group 4): A minimal blood flow signal is seen (power Doppler)

Table 2 shows the US findings of the parathyroid adenoma and lipoadenoma cases. The median tumor sizes in groups 1, 2, 3, and 4 were 16.0 mm, 19.0 mm, 11.0 mm, and 11.5 mm, respectively, with no significant differences between the groups. The prevalence of irregular nodules (20.0% to 60.0%) was not significantly different among the four groups. In 84.0% of group 1 cases, the margin was well-defined (Fig. 1a). In contrast, in groups 2, 3, and 4 associated with adipocytes, the incidence (75–80%) of nodules with ill-defined margins was significantly higher (*p* < 0.005) (Fig. 1b). Cystic components were found in 8% and 20% of the patients in groups 1 and 2, respectively. Tumors in patients in groups 3 and 4 did not contain any cystic components. Heterogeneity tended to be observed with an increasing number of adipocytes; however, no statistically significant differences were identified. Nodules in which more than 30% of the nodules was isoechoic and/or hyperechoic were present in 80–100% of cases in groups 2, 3, and 4, but only in 20% of cases in group 1 (*p* < 0.005). Nodules segmented by two different echogenic levels (two-tone pattern) were more frequently observed in the group with more adipocytes (Fig. 2a), with a frequency of 75.0% in group 4. Calcification was observed in only one patient in group 2. The calcification was 2 mm in size and associated with an acoustic shadow. Blood flow signals were present in most patients in groups 1 and 2, but not in 80% of patients in group 3 and none of the patients in group 4 (*p* < 0.0001, Fig. 3). Similarly, polar arteries and hyperechoic lines between the thyroid and tumor were frequently present in groups 1 and 2, but not in groups 3 and 4 (*p* < 0.0005 and *p* < 0.05, respectively).

**Table 2:** Ultrasonographic findings of parathyroid adenoma and lipoadenoma cases

	Adenoma			Lipoadenoma	*p-*value
	Group 1 (n = 25)	Group 2 (n = 5)	Group 3 (n = 5)	Group 4 (n = 4)	
Tumor size*, mm (median)	6–41 (16.0)	11–22 (19.0)	10–14 (11.0)	9–22 (11.5)	NS
Irregular shape	6/25 (24.0%)	1/5 (20.0%)	3/5 (60.0%)	1/4 (25.0%)	NS
Ill-defined margin	4/25 (16.0%)	4/5 (80.0%)	4/5 (80.0%)	3/4 (75.0%)	< 0.005
Solid	23/25 (92.0%)	4/5 (80.0%)	5/5 (100%)	4/4 (100%)	NS
Predominantly solid	1/25 (4.0%)	0/5 (0%)	0/5 (0%)	0/4 (0%)	
Predominantly cystic	1/25 (4.0%)	1/5 (20.0%)	0/5 (0%)	0/4 (0%)	
Cystic	0/25(0%)	0/5 (0%)	0/5 (0%)	0/4 (0%)	
Heterogeneity	4/25 (16.0%)	2/5 (40.0%)	2/5 (40.0%)	3/4 (75.0%)	NS
Iso- and/or hyper-echogenicity	5/25 (20.0%)	4/5 (80.0%)	4/5 (80.0%)	4/4 (100%)	< 0.005
Two-tone pattern	1/25 (4.0%)	1 /5 (20.0%)	2/5 (40.0%)	3/4 (75.0%)	< 0.005
Calcification	0/25 (0%)	1/5 (20.0%)	0/5 (0%)	0/4 (0%)	NS
Blood flow signal	23/25 (92.0%)	5/5 (100%)	1/5 (20.0%)	0/4 (0%)	< 0.0001
Polar artery	21/25 (84.0%)	2/5 (40.0%)	0/5 (0%)	0/4 (0%)	< 0.0005
Hyperechoic line	15/19 (78.9%)	2/4 (50.0%)	0/3 (0%)	0/1 (0%)	< 0.05

NS, not significant

### US and pathological correlations

**Fig. 4:**
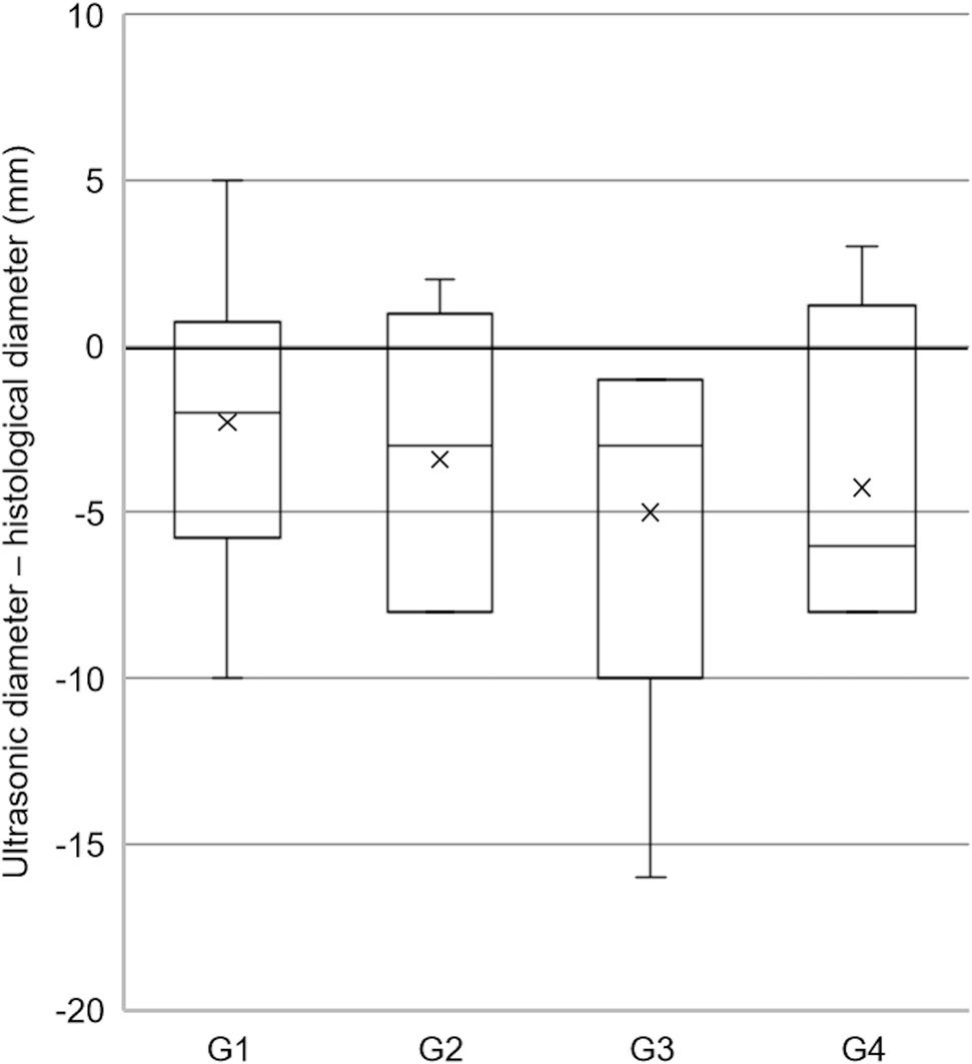
Differences in tumor size measured using ultrasound compared with the actual tumor size

**Fig. 5:**
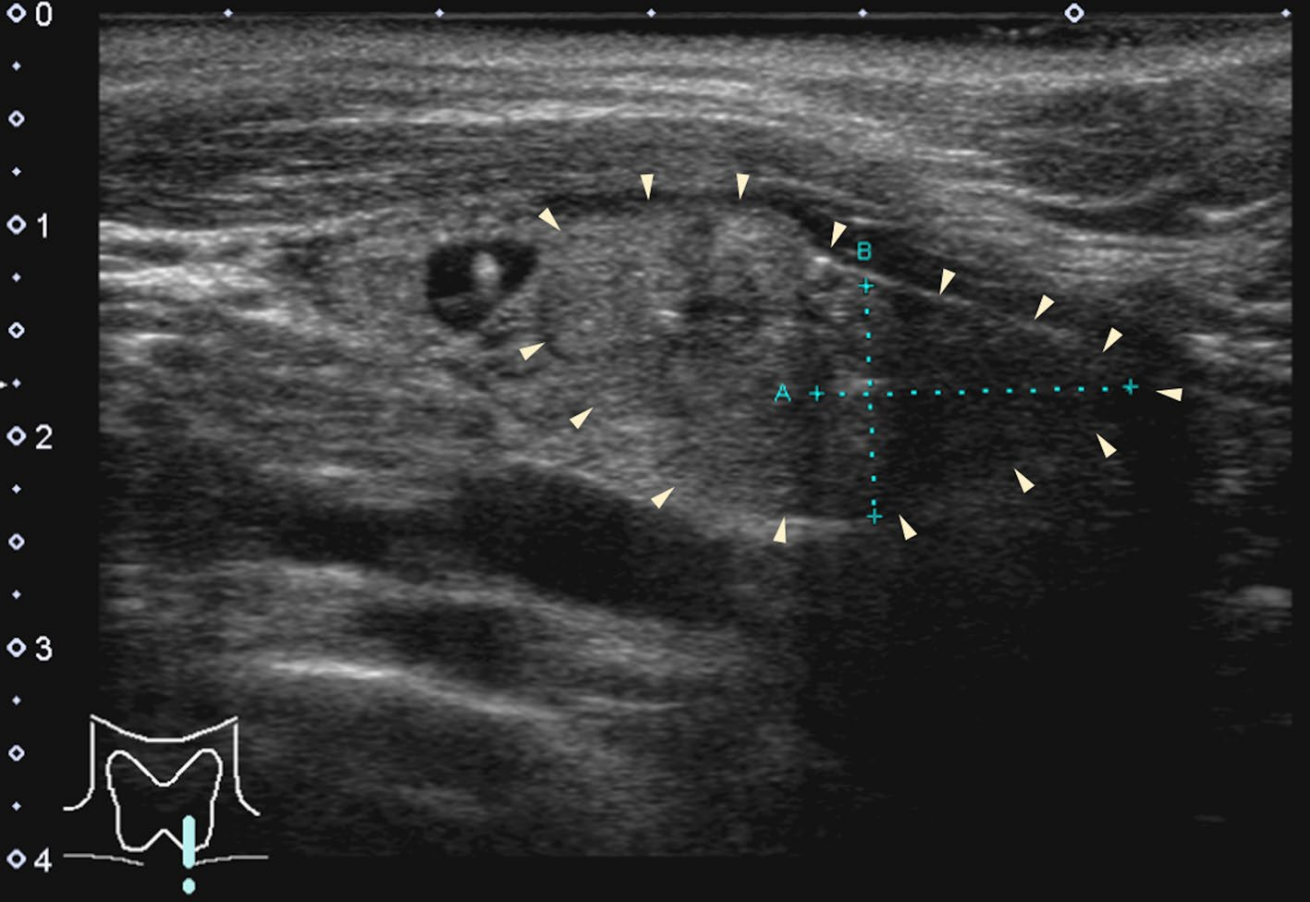
A parathyroid adenoma with heterogeneous echogenicity (Group 2). Triangular arrows indicate the margins of the tumor. On ultrasound examination, only a hypoechoic area was recognized as a tumor

In tumors presenting a two-tone pattern on US, hypoechoic and isoechoic areas corresponded to adipocyte- and parathyroid cell-rich areas, respectively (Fig. 2b). The median tumor sizes measured based on histological preparations were 18 mm, 19 mm, 14 mm, and 18 mm in groups 1, 2, 3, and 4, respectively. US measurements tended to be smaller than the actual sizes in all groups but were particularly prominent in group 4 (Fig. 4). In seven tumors with heterogeneous echogenicity, tumor sizes were measured by judging only the hypo- or iso-echogenic areas as nodules. Figure 5 shows a case with differences in tumor size between US and histological preparations.

## Discussion

Parathyroid lipoadenomas are benign tumors composed of parathyroid cells and adipocytes. Histological diagnosis is occasionally problematic because normal parathyroid glands contain at least 25% adipocytes [1]. In this study, groups 1 and 4 had typical parathyroid adenoma and lipoadenoma characteristics, respectively. However, groups 2 and 3 had intermediate fat volumes. By comparing these groups, the influence of adipocyte volume on US findings was determined.

Cervical US is recommended for the detection of parathyroid adenomas in patients with hyperparathyroidism. The diagnostic accuracy is high and the cost is lower than that of other imaging modalities [6]. However, in cases with a lipoadenoma, the detection rate is 58% [12]. In the present study, the detection rate of lipoadenomas was 20.0%, which was significantly lower than that in groups with no or fewer adipocytes, and it increased to 80.0% after obtaining information from other imaging modalities. This suggests that the localization of lipoadenomas by means of US alone is difficult; however, they can be detected to the same degree of accuracy as parathyroid adenomas if the US findings are well defined. In addition, it is unlikely that the degree of experience of the technicians affected the detection rate as the debatable cases were double-checked by senior technicians.

US findings of parathyroid adenomas are well documented. These tumors are characterized by an oval, predominantly solid, homogeneous, and hypoechoic nodule with a well-defined margin, polar artery, and hyperechoic line [7–9]. An enlarged feeding artery is also observed [9]. Parathyroid adenomas may contain a cystic area or be heterogeneous, especially when they are larger than 2 cm in size [9]. The internal vascular flow to a parathyroid adenoma varies. US findings of lipoadenomas in the literature are limited. Obara et al. reported that hyperechoic tumors are characteristic of lipoadenomas [7]. Their hyperechoic nature is due to the fatty stroma, which is the only distinction from a hypoechogenic parathyroid adenoma.

In the present study, we compared the US findings of adenomas with no or few adipocytes and lipoadenomas. Findings other than echogenicity were useful for differentiating between the two. The US findings of lipoadenomas are characterized by ill-defined margins, iso- and/or hyperechogenicity, heterogeneous consistency with a two-tone pattern, poor vascular flow, no polar artery, and no hyperechoic line. Because these findings are not features of parathyroid adenomas, they are thought to help differentiate between the two. In particular, we considered a two-tone pattern to be a significant finding suggestive of a parathyroid adenoma containing adipocytes and lipoadenoma. This may be due to the heterogeneous distribution of adipocytes. Moreover, we found that the blood flow in lipoadenomas is poorer than that in parathyroid adenomas. Previous studies have also shown that lipomas typically have a poor blood flow [19, 20]. Lipoadenomas predominantly comprise fat cells. Thus, the blood flow to a lipoadenoma is presumed to be poor relative to its size; hence, it was difficult to detect polar arteries.

Furthermore, another pitfall in the observation of lipoadenomas on US was identified. The tumor sizes of lipoadenomas on US tended to be smaller than their actual sizes. Only the hypo- or isoechogenic areas were judged to be nodules. The recognition that lipoadenomas typically have a heterogeneous consistency with a two-tone pattern may help avoid this pitfall.

A lipoadenoma containing excessive adipocytes, lipoma, and involuted ectopic thymus are considered part of the differential diagnosis for lipoadenoma. Lipomas are benign tumors composed of adipose tissue that are frequently found in the neck. On US, lipomas are oval and have varied echogenicity depending on the proportion of adipocytes and fibrous tissue. Striated echogenicity is characteristic of a lipoma [21, 22] and is helpful in distinguishing it from a lipoadenoma. An involuted ectopic thymus, called a phantom nodule, is present in the caudal region of the thyroid and appears as a well-defined, solid, homogeneous, and hyperechoic nodule [23]. In the present study, one lipoadenoma was interpreted as an ectopic thymus. An involuted ectopic thymus is usually not heterogeneous as in lipoadenoma [23]. Reactive lymph nodes may also be similar to those observed in lipoadenomas. Well-defined hypoechoic nodules with an eccentric echogenic hilum indicate reactive lymph nodes rather than a lipoadenoma [22]. The presence of hyperparathyroidism, knowledge of the US findings that are characteristic of lipoadenomas, and information on other imaging modalities may be helpful in differentiating between them.

The limitations of this study include its small sample size and retrospective nature.

In conclusion, we identified the US findings characteristic of lipoadenomas. These included ill-defined margins, iso- and/or hyper-echogenicity, heterogeneous consistency with a two-tone pattern, poor vascular flow, no polar artery, and no hyperechoic line. These findings are clearly different from those observed in parathyroid adenomas without adipocytes. We believe that focusing on these findings and referring to other imaging modalities increase the detection rate of lipoadenomas and allow us to consider them in the differential diagnosis. Future large-scale multicenter studies may be needed to further validate our findings.

## Data Availability

The data supporting the findings of this study are available from the corresponding author upon reasonable request.
